# Differences in inhibitory control in two species of Tanganyikan bower‐building cichlids contrasting in building flexibility

**DOI:** 10.1002/ece3.11406

**Published:** 2024-06-06

**Authors:** Maëlan Tomasek, Katinka Soller, Valérie Dufour, Alex Jordan

**Affiliations:** ^1^ LAboratoire de Psychologie Sociale et Cognitive UMR6024, CNRS, UCA Clermont‐Ferrand France; ^2^ Behavioural Evolution Research Group Max Planck Institute of Animal Behaviour Konstanz Germany; ^3^ University of Konstanz Konstanz Germany

**Keywords:** animal construction, cognitive flexibility, comparative cognition, lek, preference, wild animals

## Abstract

A central challenge in understanding the evolution of cognition is the ability to compare a set of species differing in a trait of interest while being ecologically and phylogenetically close. Here, we examine whether differences in bower‐building flexibility are related to differences in cognitive flexibility between two Tanganyikan cichlids. Cognitive flexibility enables animals to modify their decision rules when faced with new situations, and inhibitory control, the ability to inhibit a normally favoured response, is an essential component of this capacity. We tested male *Aulonocranus dewindti* and *Cyathopharynx furcifer* in a choice‐against‐preference paradigm. Both species clean their bowers of foreign objects and we found that both preferred to remove a snail shell over a stone. We tested their ability to modify this preference and learned to preferably select the stone instead of the shell. Although neither species showed clear learning of the new preference rule, both demonstrated inhibitory control through increased decision times and manipulations of the objects when selecting the stone. Specifically, *A. dewindti*, the species exhibiting greater behavioural flexibility in the construction of their bowers, selected the stone in fewer trials than *C. furcifer*, providing support for a link between behavioural flexibility in bower construction and cognitive flexibility.

## Graphical abstract

Behavioural flexibility in animal constructions has been hypothesized to be linked with advanced cognitive abilities. In this paper, we compare two species of Tanganyikan bower‐building cichlids and show that the species building more flexible sand bowers also demonstrated better performances in a choice‐against‐preference task assessing inhibitory control.

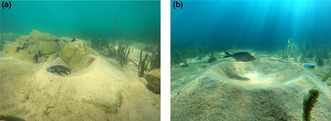

## 1 INTRODUCTION

Comparing cognitive flexibility between different species is a potentially powerful way to probe the socio‐ecological factors underlying the evolution of cognition. Cognitive flexibility is defined as the ability to modify one's decision rules and is crucial for individuals to adapt to new situations and navigate unpredictable and changing environments (Cañas et al., 2000). It can vary both qualitatively and quantitatively between species from distant taxa (Gonzalez et al., 1967; Mackintosh, 1968; Rayburn‐Reeves et al., 2013). In closely related species, differences in cognitive flexibility have been linked to habitat differences (lizards: Szabo & Whiting, 2020), foraging strategies (lizards: Day et al., 1999; bats: Clarin et al., 2013) or differences in social complexity (corvids: Bond et al., 2007; parrots: Van Horik & Emery, 2018; and cichlids: Fischer et al., 2021). Cognitive flexibility, as part of a suite of more complex cognitive skills, can also be linked to greater behavioural flexibility (Mikhalevich et al., 2017). In nest‐building behaviour, for example, some authors have suggested a link between nest‐building complexity and the neural underpinning of advanced cognitive skills (Guillette & Healy, 2015). This type of relationship has also been observed in other types of construction, for example, bowerbird species that build more complex bowers possess larger brains (primarily cerebella) (Day et al., 2005; Madden, 2001). Note, however, that species contrasting in brain size and bower complexity may not always show different performances in cognitive tasks (as shown in a problem‐solving task in two bowerbird species: Isden et al., 2013; Keagy et al., 2009, 2011, 2012). Nevertheless, these results suggest that building more complex nests, in a more flexible way, could be linked with greater cognitive flexibility, and nest or bower‐building flexibility could thus be a good marker of cognitive flexibility. According to Mikhalevich et al. (2017), behavioural flexibility can serve as evidence for underlying complex cognitive mechanisms (e.g. cognitive flexibility), when the weight of surrounding biological variables (evolutionary, ecological and phylogenetic) has been given due consideration.

While research on animal construction has mostly focused on birds, there are other taxa that build structures of differing complexity and of these, fish can be good models in which the link between complex construction and cognitive flexibility can be investigated. Indeed, fish represent over 50% of extant vertebrates, and their cognitive flexibility has been studied in various species with a diversity of paradigms testing for inhibitory control. Inhibitory control, the ability to inhibit a normally favoured response, is an essential component of cognitive flexibility (Beran, 2018; Izquierdo et al., 2017). In detour tasks, where fish must learn to swim around a transparent barrier to access a reward, cichlid fish have performed successfully (Brandão et al., 2019; Jungwirth et al., 2024; Salena et al., 2022). In another paradigm, the reverse reward contingency task, individuals must learn to choose the least preferred reward out of two alternatives in order to obtain the most desired one. Among fish species, only cleaner wrasses (*Labroides dimidiatus*) have been tested in this task to explore the inhibitory control abilities that they exhibit in the wild. Indeed, in the interactions with their clients, they often refrain from picking on their clients' mucus and eat instead of their ectoparasites, which is a less preferred option (Grutter & Bshary, 2003). Yet, they failed a reverse reward contingency task conducted in controlled laboratory conditions (Danisman et al., 2010). Finally, reversal learning tasks are the most commonly used paradigms, in which cleaner wrasses, as well as some cichlids, show good performances (Aellen et al., 2022; Culbert et al., 2020, 2021; Fischer et al., 2021; Ingraham et al., 2016; Salena et al., 2022; Triki & Bshary, 2021).

**FIGURE 1:**
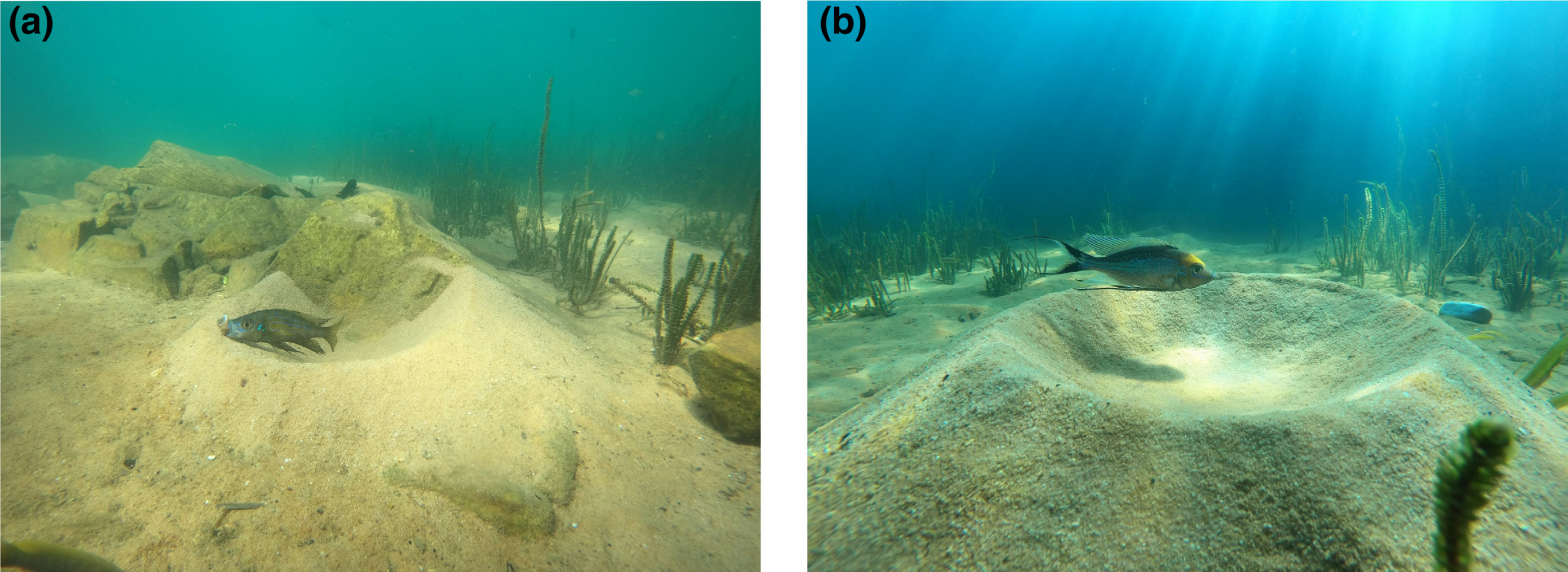
Typical bower structures of (a) *Aulonocranus dewindti* (removing a snail shell from its bower), with a quicker and more flexible bower construction integrated into the rocks present in the environment; and (b) *Cyathopharynx furcifer*, with a slower and less flexible bower construction only on flat surfaces.

Cichlids are a particularly interesting group to study as they construct a wide variety of nests or bowers. Two species from Lake Tanganyika, *Aulonocranus dewindti* and *Cyathopharynx furcifer*, build different forms of bowers and are phylogenetically close, the last common ancestor is estimated at <2 million years ago (Ronco et al., 2021). This closeness reduces phylogenetic noise as a potential cause of interspecific variability in cognitive flexibility (MacLean et al., 2012). They are also ecologically similar, living in the same habitat and sharing a similar social structure (a lekking system comparable to bowerbirds). Additionally, although *A. dewindti* is an open‐water plankton feeder, and *C. furcifer* is a sand‐filtering algae feeder, both species use passive foraging strategies, a foraging style that is not particularly demanding in terms of behavioural flexibility (Clarin et al., 2013). However, the two species differ in the way they build their bowers (Figure 1). On the one hand, *C. furcifer* shows low flexibility in their constructions. They build sandcastles, either on open sand or on a relatively flat rock or hard surface. Bowers can reach considerable heights (more than 50 cm) and the construction is a slow process that can last up to several days (Schaedelin & Taborsky, 2006). In this species, bower construction shows little intra‐individual variability as each bower crater is individually specific in size and each individual will rebuild a crater of the exact same size after damage (Schaedelin & Taborsky, 2006). By contrast, the less studied *A. dewindti* shows greater flexibility when building their bowers. As in *C. furcifer*, they can build bowers on open surfaces, but they can also flexibly incorporate more complex features of the environment, for example, larger rocks, into their constructions. While the bower itself is circular, one or several rock faces can be flexibly incorporated into the design, for example, a semi‐circle against a single rock face, or a bower ‘wrapped’ around an angular rock face (MT, personal observations; see also Konings, 1998, p283). In *A. dewindti*, the construction is quick (usually a few hours) and on several occasions, we observed some males building an entirely new bower, whereas this observation was never made for *C. furcifer*. This difference in bower building flexibility may be an indicator of overall behavioural flexibility differences among the species. Reproductive behaviours may also be indicative of lower behavioural flexibility in *C. furcifer* than in *A. dewindti*. *C. furcifer* males seem to only be ‘bower‐holding males’ or males waiting to occupy a deserted bower (Karino, 1996), whereas *A. dewindti* males have been described as ‘bower‐holding males’, ‘sneaker males’ (do not own a bower but try to fertilize the eggs during the reproductive act of another bower‐holding male), ‘pirate males’ (attack other bowers to temporarily take control of them) or ‘floating males’ (wander around to find unoccupied bowers) (MT, personal observations; Konings, 1998, p284). Note, however, that reports of these differences remain anecdotal and given the lack of scientific evaluation of these reproductive behaviours, it is not clear whether the same individual can hold several roles in *A. dewindti*.

Being in a lekking system, both species are highly motivated to maintain their bowers and remove any unwanted foreign object falling into them. This motivation makes them suitable candidates for a comparison of their performances in a choice‐against‐preference task developed in a previous study (Tomasek et al., 2023). In this earlier study, we investigated cognitive flexibility in wild *A. dewindti*. We first observed that when a snail shell and a stone were placed together in the bower at the same time, *A. dewindti* predominantly removed the snail shell first. We took advantage of this spontaneous preference to investigate whether males could modify this preference rule and learn to remove the stone first. To do so, each time they removed the shell first, we continuously replaced it in the bower, thereby increasing the effort associated with that object when cleaning the bower. If they removed the stone first, we did not replace it in the bower and allowed them to remove the remaining shell without any interference from us. Some individuals showed inhibitory control, while it was harder for other individuals to resist the drive to remove the shell first. In our task, however, producing the correct answer (removing the stone first) still required them to come back to finish cleaning the bower and remove the shell. Thus, instead of learning a simple association, they had to learn an ordinality rule (first remove the stone and then remove the shell) which renders the task more difficult. Here, we used a modified version of this paradigm to compare cognitive flexibility between wild *A. dewindti* and *C. furcifer*. First, we investigated whether the preference rule to remove the shell first when both shell and stone were placed in the bower was also present in *C. furcifer* and confirmed in *A. dewindti*. Second, in the choice‐against‐preference task, both objects were attached by a transparent thread so that although only one object was selected, both were removed from the bower. If the individual chose to remove the shell, both objects were placed back in the bower until they selected the stone. If building flexibility is connected to cognitive flexibility in bower‐building cichlids, we should observe better performances in *A. dewindti* compared to *C. furcifer* in this task.

## 2 PHASE 1: PREFERENCE TASK

### 2.1 Materials and methods

#### 2.1.1 Subjects

Adult male *A. dewindti* and *C. furcifer* were found along the south‐eastern Zambian shore of Lake Tanganyika (Isanga Bay 8°37′24.7″ S, 31°12′02.9″ E) at depths between 3 m and 5 m. All tested *A. dewindti* held bowers which were against a rock face, while all bowers of *C. furcifer* were on open sand. Nine *A. dewindti* and seven *C. furcifer* were tested, however, one *A. dewindti* became unresponsive and did not complete the task. An individual was considered unresponsive when it did not remove objects that were placed in the bower within 5 min. Responsive individuals typically removed items within seconds. Individuals could be recognized from day to day because bower‐holding males are highly philopatric and so were at the same bower each day (Schaedelin & Taborsky, 2006). They could also be further distinguished based on physical differences (size, body markings and unvarying colour hues). Experiments were conducted while scuba diving and took place between 06:00 and 11:00 AM between April 16th and May 13th, 2023.

#### 2.1.2 Preference task procedure

For each trial, an experimenter placed an empty *Lavigeria grandis* snail shell and a stone in the bower of the focal individual. The trial ended when the fish had removed both objects from its bower (Video 1). The trials were video recorded with a GoPro7 at 25fps. Different shells and stones were used, but each individual was tested with the same pair of shells/stones which were controlled to be of approximately the same weight. This resulted in the stones being slightly smaller than their paired shells (shell diameter: 2.36 ± 0.25 cm, shell weight: 1.68 ± 0.42 g, *n* = 5; stone diameter: 1.58 ± 0.28 cm, stone weight: 1.62 ± 0.17 g, *n* = 5). Fish could have then based their choice on the relative size of the objects (preferring a priori the larger one). Future experiments aim at exploring the exact cause of these preferences, but for the purposes of this study, it is important and sufficient that both species show a strong preference for removing one object over the other. Both shells and stones were selected to be easily removed by every individual. Hence, *A. dewindti* were exposed to relatively smaller pairs of shells and stones than *C. furcifer*, which are larger fish.

**VIDEO 1:** Illustration of the experimental procedure: preference task and choice‐against‐preference task with training stage (exposure) and test stage.

Every fish was tested for six sessions of five trials in a row (amounting to a total of 30 trials). The sessions were either conducted on the same day (for three *A. dewindti* and two *C. furcifer*) or across several days (all remaining individuals), which did not appear to have an impact on the preference results. Based on the results of exact binomial tests, if an individual removed the shell first more than 20 times in the 30 trials, this individual was considered to show a preference to remove the shell first. If it did so fewer than 20 times, then it was considered to not show any preference. One *C. furcifer* removed the shell exactly 20 times which was an ambiguous response. To confirm or reject the existence of a preference in this individual, we conducted 10 additional trials, which confirmed such a preference (see below). In each trial video, we scored which object was removed first and the time to that decision, defined as the time between the individual ‘seeing’ the objects in the bower (either when the fish returned and crossed the rim of the bower or when the fish clearly switched direction to swim towards the bower, while being at a height enabling him to see inside) and the removal of the first object (the object crossing the rim of the bower).

#### 2.1.3 Statistics

All statistical analyses were done using R (version 4.2.2). A two‐tailed exact binomial test was performed to test preference for all individuals (null hypothesis set at 50%, ‘stats’ package in R). Additionally, we investigated interspecific differences in the distributions of the preferred objects removed first in individuals showing a statistically significant preference for removing the shell first which later completed phase 2 (six *A. dewindti* and five *C. furcifer*). We removed the *C. furcifer* individual which did 10 more trials than the others because its response in the task could be driving the results. We ran a generalized linear mixed‐effect model (Object removed first ~ Species + (1|Subject)) with a binomial family distribution (package ‘glmmTMB’ in R; Brooks et al., 2017). For each generalized linear model, we analysed the residuals to explore the fit of the models (‘DHARMa’ package in R; Hartig, 2017). To investigate differences in decision time depending on the species or the object removed, we ran a Cox regression model (Decision time ~ Species*Object removed first + (1|Subject)) (package ‘coxme’ in R; T. M. Therneau, 2015). For each Cox regression model, we checked for proportional hazards with Schoenfeld tests (function ‘cox.zph’, package ‘survival’ in R; T. Therneau & Lumley, 2015). We then ran a type II ANOVA to analyse the contribution in the variance of each factor (package ‘car’ in R; Fox & Weisberg, 2019).

### 2.2 Results

**FIGURE 2:**
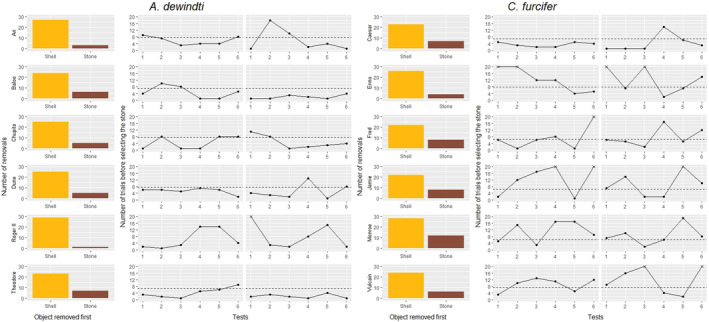
Results of the preference task and the choice‐against‐preference task for each individual of each species. Note that only the six individuals that participated in the choice‐against‐preference task are represented (see main text). The bar plots are the individual results of the preference task (which object had been removed first from the bower). The preferences are statistically significant for all individuals (*p* < .05, exact binomial test, see main text and Table S1A). Next to the bar plot, the results of the choice‐against‐preference task corresponding to the same individual are represented. The two grey panels are the two different sessions (left: session 1; right: session 2). Each session is composed of six tests. The y‐axis is the number of trials taken by the individual to select the stone in each test (dots: tests in which the individual finished by selecting the stone; crosses: tests in which the individual selected the shell 20 times in a row and the test was ended). Tests above the dashed line had <5% chance of happening should the selection choices follow the preferences displayed in the preference task only (see main text and Supporting Information). This line is adapted to individual results in the preference task. Note that there is no line for Roger II (*Aulonocranus dewindti*) because the probability of selecting the stone in its preference task was 3%, which is under the alpha‐level of 5% used in this statistical analysis (there is already <5% chance that this individual selects the stone on the first trial).

Almost all individuals showed a significant preference for removing the shell first: seven of eight individuals in *A. dewindti* and six of seven individuals in *C. furcifer* (Figure 2, Table S1A). These individuals were then investigated for their cognitive flexibility in the choice‐against‐preference task (phase 2).

**FIGURE 3:**
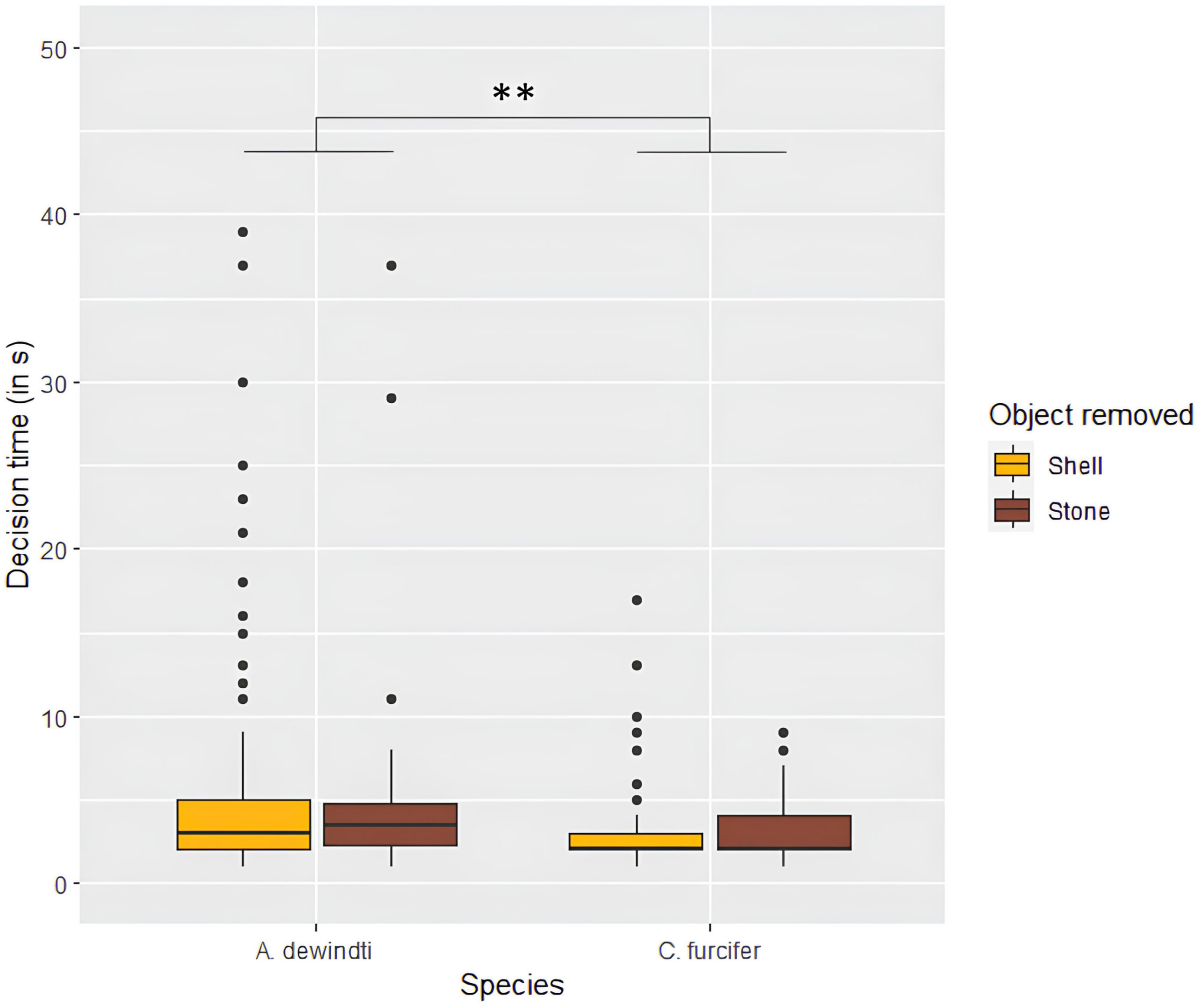
Decision time to remove the first object in the preference task (**p < .01, ANOVA from cox regression mixed‐effect model) (Note that two values above 50s are not shown for *Aulonocranus dewindti*).

There was no significant difference in the distribution of the preferences between species (estimate = 0.46 ± 0.29, *p* = .10; Table S1B). There was a significant difference in decision times between the species (ANOVA *χ* ^2^ = 7.95, *p* < .01; Figure 3, Table S1C) with *A. dewindti* taking longer to remove the objects than *C. furcifer* (mean decision time *A. dewindti* = 9.0s, mean decision time *C. furcifer* = 2.9s).

## 3 PHASE 2: CHOICE‐AGAINST‐PREFERENCE TASK

### 3.1 Materials and methods

#### 3.1.1 Experimental procedure

**FIGURE 4:**
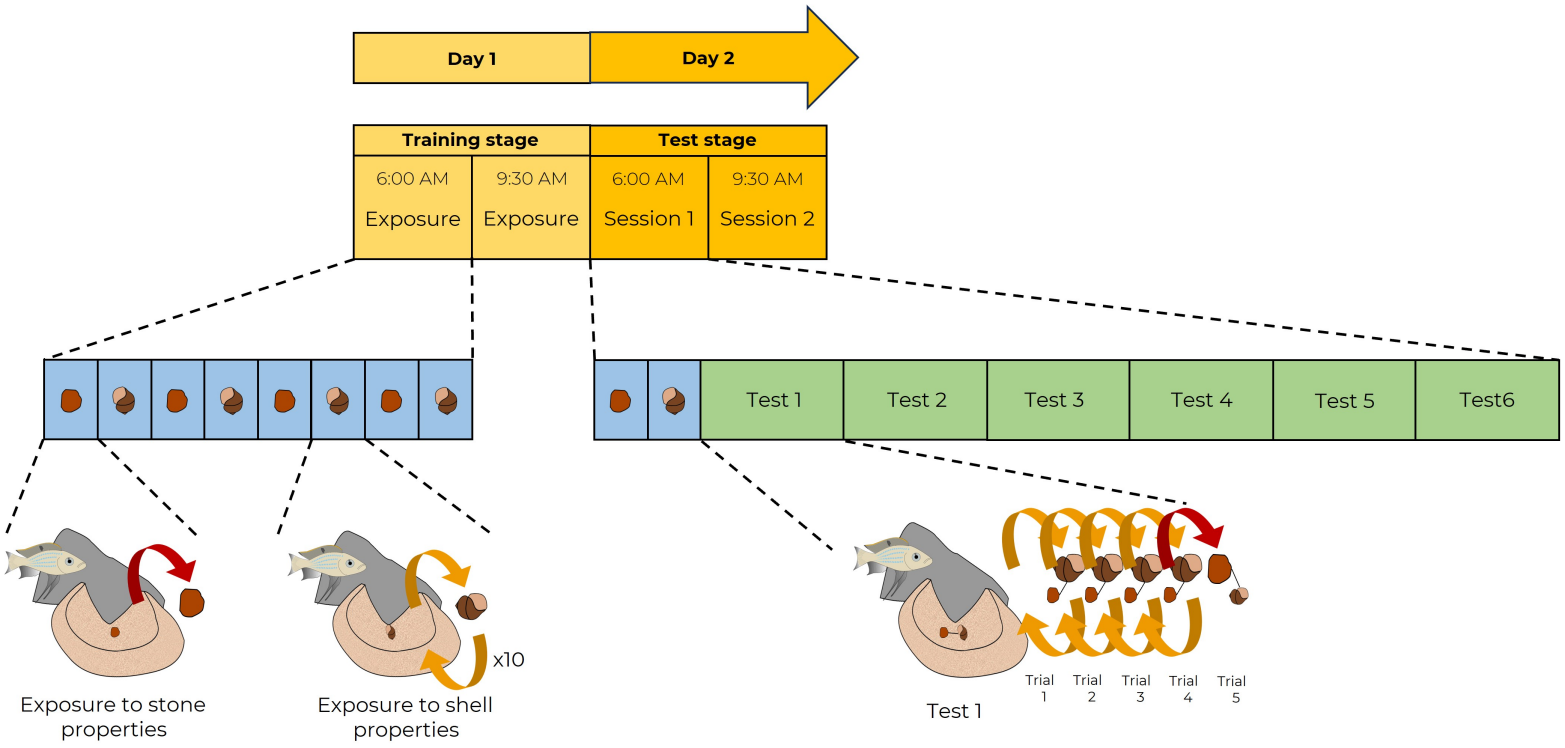
Experimental procedure of the choice‐against‐preference task (phase 2). On the first day, in the training stage, fish went through two exposure sessions. In those sessions, they were presented alternatively with the stone or the shell. When the stone was put in their bower, the fish could remove it and the experimenter would swim away. When the shell was put in their bower, the fish could remove it, but the experimenter placed it back in the bower, a total of 10 times. On the second day, in the testing stage, we conducted two sessions of the choice‐against‐preference task per se. In each session, the experimenter first presented every object individually once as during the exposure sessions. Then, six tests of the cognitive task were conducted. In each test, a shell and a stone that were bound by a transparent thread were put in the bower. If the fish removed the objects by picking up the shell, both objects were immediately put back in the bower to start a new trial. If the fish removed the objects by picking up the stone, the experimenter ended the test, retrieved the objects and swam away. In this example, it takes the fish five trials to select the stone. See main text for more details.

To enter this phase, individuals had to show a preference for removing the shell first in phase 1. One of the seven *A. dewindti* successful in phase 1 was dropped out in phase 2 because he became unresponsive, so a total of six fish for each species completed this phase. Phase 2 was divided into two stages, which occurred on 2 consecutive days: the training stage with the exposure to the properties of the objects (day 1) and the testing stage with the choice‐against‐preference task per se (day 2, Video 1, Figure 4). Once again, shells and stones used in this phase were controlled for approximate same size and weight (shell diameter: 2.17 ± 0.23 cm, shell weight: 1.79 ± 0.44 g, *n* = 6; stone diameter: 1.58 ± 0.28 cm, stone weight: 1.68 ± 0.12 g, *n* = 6).

During the training stage of phase 2 (day 1), the individuals were exposed to the differing properties of the stone and the shell one object at a time. For example, fish were first exposed to a stone that the experimenter placed in the bower. Once they removed the stone, the experimenter swam away for at least 3 min. Thus, fish had the opportunity to learn that removing the stone was a successful action leading to the disappearance of the experimenter and of the objects coming into their bower, both of which may deter visiting females. We consider fish to interpret this as a beneficial outcome as their bower becomes attractive again. Then, the experimenter came back and placed a shell in the bower. When the fish removed the shell, the experimenter immediately placed the shell back in the bower. The fish had to remove the shell 10 times for the experimenter to swim away for at least 3 min. Here, the fish had the opportunity to learn that removing the shell was not a successful action (it keeps coming back). Presentations of the stone (which did not come back) and of the shell (which came back 10 times) were alternated, and the order of the objects (first presented with the stone or first presented with the shell) was randomized across individuals. Each condition with each object was presented four times within a session for a total of two sessions (the first session was conducted between 6:00 and 8:00 AM, and the other between 9:30 and 11:30 AM on the same day). This meant that in a day, fish were presented in total eight times with the stone (which did not come back) and eight times with the shell (which came back 10 times). All individuals were exposed to the same number of presentations.

For the testing stage of phase 2 (day 2), before each session, we first presented the individuals with each single object once to remind them of the properties of the objects (the stone that does not come back and the shell that comes back 10 times). The order of presentation was randomized across individuals. Then the choice‐against‐preference task per se began. A test was composed of several trials (from 1 to 20 trials). For each trial, the experimenter placed in the bower a shell and a stone that were bound by a transparent fishing line. Thus, whichever object was chosen and transported by the fish, both were automatically removed from the bower. The trial ended when the object that the fish selected (took in its mouth and removed from the bower) crossed the rim of the bower. If the fish selected the stone, the experimenter took both objects, swam away and the test was ended. If the fish selected the shell, both bound objects were placed immediately back in the bower and a new trial began in the same test until the fish selected the stone. If a fish selected the shell in 20 consecutive trials, the test was also ended. One test could therefore last from 1 trial (if the fish selected the stone in trial 1) to 20 trials (if the fish never selected the stone). Two sessions of six tests were conducted for each individual on the same day (session 1: between 6:00 and 8:00 AM, session 2: between 9:30 and 11:30 AM). Tests were separated by at least 3 min during which the experimenter stayed away from the bower.

For each test, we recorded the number of trials needed to remove the stone (note that if a fish selected the shell 20 times in a row, it was scored as ‘20’ even though the individual actually never selected the stone). We scored the decision times (as defined earlier) as well as the manipulations of the objects inside the bower (defined as the actions oriented towards the objects before removing one from the bower, such as touching or nudging an object, see Tomasek et al. (2023)).

#### 3.1.2 Statistics

##### Overall performance

We investigated whether the fish had changed their preferences towards the objects compared to the preference task (phase 1). The experimental procedure was asymmetric (selecting the stone ends the task) and standard tests designed to compare proportions could not be used because the fish did not have the same number of opportunities to select both objects. Nevertheless, assuming a geometric distribution, we could test whether an individual had increased its preference to select the shell compared to the preference task. To do so, we calculated a threshold number of trials that would be statistically unlikely (<5% chance) to be reached if initial preferences shown in the preference task stayed unchanged. For all individuals, this threshold was between six and eight trials. Requiring more than this number of trials to select the stone would therefore be interpreted as an individual having increased its preference for selecting the shell compared to phase 1. Note that if this threshold was not crossed, we could not distinguish if the individual had significantly increased their preference to select the stone or if the preferences had remained unchanged. To do so, we could only rely on behavioural indices (see Section S2 for an extensive explanation of this test).

We investigated interspecific differences in the general success of the task (number of trials needed to select the stone). We also investigated whether there was a change of performance over time: between session epochs (between the first and the second sessions) or between test epochs (between the beginning tests, i.e. tests 1, 2 and 3, and the end tests, i.e. tests 4, 5 and 6; Tomasek et al., 2023). We ran a generalised linear mixed‐effect model (Number of trials needed to select the stone ~ (Session epoch + Test epoch)*Species + (1|Subject)) with a negative binomial distribution. An analysis of the model residuals and a type II ANOVA were conducted as mentioned above.

##### Behavioural differences

As in our previous study (Tomasek et al., 2023), we investigated two behavioural indices to further assess the comprehension of the task: the decision time and the number of object‐oriented actions (‘manipulations’) inside the bower before making a choice. A longer decision time and more manipulations of the objects before making a choice were interpreted as a less automatic response (Tomasek et al., 2023).

To investigate differences in decision time, we ran a Cox regression model (Decision time ~ Species*Object selected + (1|Subject)). To investigate manipulations, we ran a generalized mixed‐effect model (Number of manipulations ~ Object selected*Species + (1|Subject)) with a Poisson family. For both models, model analyses were done as described above and, if relevant, post hoc analyses were conducted (function ‘lsmeans’, ‘lsmeans’ package in R; Lenth, 2016).

### 3.2 Results

#### 3.2.1 Overall performance

**FIGURE 5:**
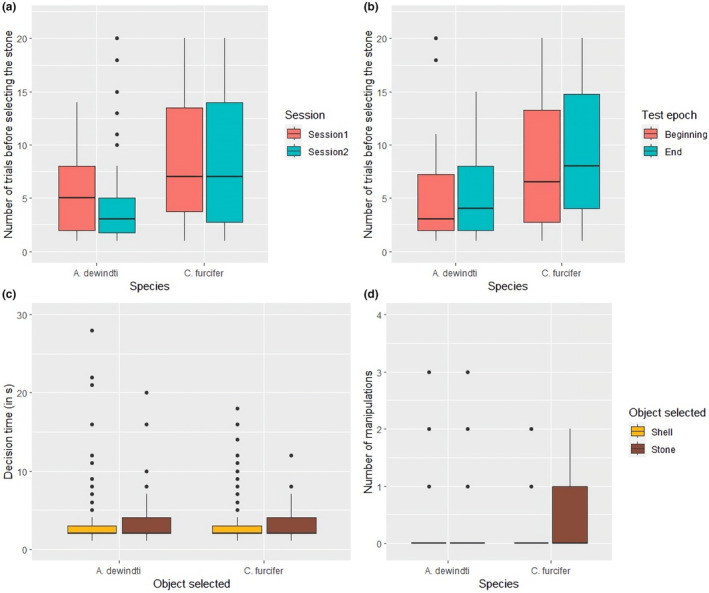
Performance of each species in the choice‐against‐preference task (see main text and Supporting Information for detailed statistical results). (a) Overall success in the task between sessions. (b) Overall success in the task between test epochs. (c) Decision time to select an object (please note that two data points have not been represented for clarity, one at 66s and the other at 73s, both for *Aulonocranus dewindti* selecting the shell). (d) Number of manipulations of the objects inside the bower before selecting an object (see Table S3B for absolute numbers).

All individuals performing at least one test that had <5% chance of occurring if they followed the preferences shown in the preference task (i.e. more than 6, 7 or 8 trials before selecting the stone, depending on the individual) (Figure 2). In these tests, their preference for selecting the shell therefore significantly increased compared to the preference task. There was a significant difference in the overall success of the task between species (ANOVA *χ* ^2^ = 9.25, *p* < .01; Figure 5, Table S2). *C. furcifer* needed more trials to select the stone than *A. dewindti* (mean number of trials *A. dewindti* = 5.0 and mean number of trials *C. furcifer* = 9.0).

#### 3.2.2 Behavioural differences

Decision times differed significantly between the two objects (ANOVA *χ* ^2^ = 9.44, *p* < .01; Figure 5c, Table S3A) with fish taking longer to select the stone than the shell (mean decision time shell = 2.8s, mean decision time stone = 3.3s).

The number of manipulations of the objects inside the bower before making a decision differed significantly between the two objects (ANOVA *χ* ^2^ = 38.33, *p* < .001) and there was also a significant effect of the interaction between species and object selected (ANOVA *χ* ^2^ = 8.56, *p* < .01; Table S3C). Fish manipulated the objects more when they selected the stone than when they selected the shell, and this effect was stronger in *C. furcifer* (Figure 5d, post hoc lsmeans test estimate = −1.59 ± 0.25, *p* < .001, Table S3D).

## 4 DISCUSSION

In the initial preference task, almost all *A. dewindti* and *C. furcifer* showed a significant preference for removing the shell first when both shell and stone were placed in their bower. In the subsequent choice‐against‐preference task, no fish appears to have completely mastered the rule ‘nothing comes back if I select the stone’. However, they behaved differently when they selected the stone (they took a longer time to decide and manipulated the objects more frequently) which suggests some inhibitory control rather than a more automatic response based on preference. The two species performed differently in this task, with *C. furcifer* needing more trials to select the stone compared to *A. dewindti*, which suggests interspecific differences in inhibitory control.

The first question that arises is whether the fish used rule‐learning mechanisms when they selected the stone. The fish had the opportunity to learn the following rule: ‘selecting the stone prevents both objects from coming back in the bower’, which was more efficient than their initial preference rule (selecting the shell), in which both objects immediately returned for up to 20 trials in the bower. If individuals had learnt the efficient rule, we would expect them to demonstrate a perfect pattern of success, that is, selecting the stone on the first trial at each test, at least in session 2. Most fish of both species required several trials before selecting the stone, including in session 2. In two *A. dewindti* individuals (Baloo and Theodore), the mean number of trials was close to 1, with a maximum of four trials per test in the second session. However, given this low number of successful individuals, evidence about rule‐learning ability here remains weak at best. Most, if not all, fish failed to understand clearly that selecting the stone was a more efficient strategy than selecting the shell.

Still, the performance of the fish does not appear to be an automatic removal of objects without any cognitive process involved either. Two types of responses were observed. In some fish, the preference for the shell became significantly higher than predicted by their initial preferences. This was particularly the case for most *C. furcifer* individuals, which needed a mean of nine trials before selecting the stone (an average increase of two to three trials compared to their initial preference). We suggest that the constant return of the objects in the bower increased their attention towards the shell, which may have made it harder for the fish to overcome the drive to remove it. By contrast, other individuals successfully selected the stone in fewer trials, especially in session 2 (all *A. dewindti* except one, and one *C. furcifer*), suggesting improved inhibitory control to overcome the drive to remove the shell. Finally, selecting the stone took longer and occurred after manipulating the objects more often, suggesting inhibitory control processes and potentially some degree of comprehension that selecting the stone brings a different outcome than selecting the shell. These results are similar to those observed in an initial and more complex version of this task (Tomasek et al., 2023) conducted on different individuals of *A. dewindti*, where fish had to acquire a more complex ordinal rule to stop the shell from coming back. In both studies, rule learning seems just out of reach of the fish, at least using these paradigms, but several individuals are nonetheless able to exert inhibitory control and increase their choice of the most efficient solution.

Our results are reminiscent of another task exploring cognitive flexibility: the reverse reward contingency task, in which an animal is given the choice between two amounts of reward and must point to the smallest amount to receive the largest. This demands strong inhibition and almost all species tested, including chimpanzees, fail in this task. Modifications of the standard protocol like the use of symbols can lead to an improvement in performance (Beran, 2022; Boysen & Berntson, 1995). Like the reverse reward contingency task, our paradigm could require a higher level of inhibitory control than most cognitive flexibility tasks (e.g. reversal learning or detour tasks). It would, therefore, be informative to conduct a task similar to ours in other species to better comprehend which cognitive mechanisms are needed to solve it and to refine what could be expected as a ‘good’ performance across different species. A group of species that seems amenable to such experiments is the bowerbird family. Indeed, these birds show strong dislikes for some colours that they would want to remove from their bower area (Endler & Day, 2006; Frith & Frith, 2008). This behaviour has been used in previous cognitive experiments but none testing cognitive flexibility, although one species has been tested in a classical reversal learning paradigm using food rewards (Isden et al., 2013; Keagy et al., 2009, 2011, 2012). We acknowledge our paradigm is open to improvements. First, in the training stage, fish are more often exposed to the shell than to the stone, which leads to different amounts of experience with each object. However, this training did not seem to improve their understanding of the properties of each object. Second, the fact that an object consistently returns to the bower might have been difficult for the fish to grasp. Future studies could also use other object properties, like different weights, to change the relative cost of moving the objects. Finally, learning might be improved by a more salient positive feedback for the correct response and future studies should aim at providing reinforcements that are perceived as better rewards than the simple disappearance of the experimenter.

The main result of this study remains the strong interspecific differences in task performance. Almost all *C. furcifer* (except one individual) needed more trials to select the stone than *A. dewindti* in the second phase of the experiment. Hence, although it is possible that both species acquired the same level of comprehension of the task, *C. furcifer* appears to show less inhibitory control than *A. dewindti*. It could be argued that the differences in performance could be explained by non‐cognitive factors such as those already demonstrated in other studies with other species (Stanton et al., 2021; Szabo et al., 2020). First, there could be a difference in the motivation to clean the bower. However, both species seemed equally motivated to do so as they did not differ in the time taken to remove the objects from their bower. Second, there could be a difference in individual experience, *A. dewindti* being more exposed to snails that are usually on rocks than *C. furcifer*. However, snails can also navigate on the sand and end up in *C. furcifer* bowers. Third, there could be a difference in the salience of the objects for each species. However, all evidence shows that both species strongly dislike the shell and appear to treat both objects similarly (as objects that need to be removed from the bower). Finally, there could be species differences in how they deal with ambiguous situations. The observation that *A. dewindti* usually took longer to select objects than *C. furcifer* could be an expression of a stronger ambiguity aversion. However, this longer delay was only detected in the preference task and is thus unlikely to explain responses in the choice‐against‐preference task. Additional tests are needed to investigate this point further.

In these two closely related species, behavioural flexibility in bower construction appears to be a good marker of cognitive flexibility. This result is, therefore, consistent with the proposed link between bower complexity and cognitive ability in bowerbirds (Madden, 2001). The ensuing question is now to understand the socio‐ecological factors that may underpin the difference in cognitive flexibility during these species' evolutionary history, points on which we can only speculate. One possibility is that increased predation risk on the smaller‐bodied *A. dewindti*, or differences in prey availability, may have caused them to seek refuge in shallower areas, consequently forcing them to adapt their bower‐building skills to more rocky environments. In these shallow areas, water movements may have been greater, which may have led to *A. dewindti* building bowers against rocks as some form of protection but also having to more frequently rebuild bowers after storms. In contrast, the deeper water species may have time to build more massive bowers due to the lack of water disturbance, with the consequence that they became less flexible in their construction behaviour. The species studied here live in close association with several other species that also build similar bowers (from the genera *Ophthalmotilapia* or *Callochromis*), allowing a wider scope of species, as well as a larger variety of tests, to be conducted in future studies to further examine the link among cognition, structural complexity and environmental selective pressures. Even more broadly, bower or nest construction is widespread across cichlids in other environments, from shell beds in Lake Tanganyika to sandy plains in Lake Malawi (Konings, 1998; Lein & Jordan, 2021; York et al., 2015), and would be interesting to investigate further, bearing in mind that comparing between species that are phylogenetically distant often leads to a wider range of potential factors explaining cognitive variability (phylogenetic noise, sociality, habitat, etc,). Nevertheless, the incredible diversity of construction behaviours across these species could help shed light on the evolutionary pressures involved in cognitive and behavioural flexibility. Cichlids and their adaptive radiation thus represent a fruitful avenue of research for future comparative cognitive studies.

## AUTHOR CONTRIBUTIONS

**Maëlan Tomasek:** Conceptualization (equal); data curation (lead); investigation (equal); methodology (equal); visualization (lead); writing – original draft (lead). **Katinka Soller:** Investigation (equal). **Valérie Dufour:** Conceptualization (equal); methodology (equal); supervision (equal); validation (equal); writing – review and editing (equal). **Alex Jordan:** Funding acquisition (lead); project administration (lead); resources (lead); supervision (equal); validation (equal); writing – review and editing (equal).

## FUNDING INFORMATION

This research was funded by the Behavioural Evolution Research Group and the Department for the Ecology of Animal Societies, Max Planck Institute of Animal Behaviour.

## CONFLICT OF INTEREST STATEMENT

The authors declare no competing interests.

## Supporting information


Data S1.

## Data Availability

Data and R script are available as Supporting Information.
