# Supplementary material for: Differences in inhibitory control in two species of Tanganyikan bower‐building cichlids contrasting in building flexibility
Source: Ecol Evol. 2024 Jun 6;14(6):e11406. doi: 10.1002/ece3.11406 (PMC11154817; doi:10.1002/ece3.11406)
Supplement: Supplementary file 1 — Data S1. [file ECE3-14-e11406-s001.zip › SupplementaryMaterialsBB2024.docx]

**Supplementary materials**

**PHASE 1: PREFERENCE TASK**

**SECTION 1: Preference task results**

**Supplementary table 1A.** Results of the preference task with p-values, estimated probabilities of removing the shell first, and confidence intervals from exact binomial tests.

| **Species** | **Individual** | **Removals shell first** | **Removals stone first** | **p-value exact binomial test** | **Probability of removing the shell first** | **Min CI (95%)** | **Max CI (95%)** |
| --- | --- | --- | --- | --- | --- | --- | --- |
| *Aulonocranus dewindti* | Ari | 27 | 3 | **8.4e-6** | 0.90 | 0.73 | 0.98 |
|  | Baloo | 24 | 6 | **1.4e-3** | 0.80 | 0.61 | 0.92 |
|  | Chupito | 25 | 5 | **3.2e-4** | 0.83 | 0.65 | 0.94 |
|  | DK1 | 16 | 14 | 0.86 | 0.53 | 0.34 | 0.72 |
|  | Dune | 25 | 5 | **3.2e-4** | 0.83 | 0.65 | 0.94 |
|  | Naps | 26 | 4 | **5.9e-5** | 0.87 | 0.69 | 0.96 |
|  | Roger II | 29 | 1 | **5.8e-8** | 0.97 | 0.83 | 1.0 |
|  | Theodore | 23 | 7 | **5.2e-3** | 0.77 | 0.58 | 0.90 |
| *Cyathopharynx furcifer* | Caesar | 23 | 7 | **5.2e-3** | 0.77 | 0.58 | 0.90 |
|  | Enno | 26 | 4 | **5.9e-5** | 0.87 | 0.69 | 0.96 |
|  | Fred | 22 | 8 | **1.6e-2** | 0.73 | 0.54 | 0.88 |
|  | Jamie | 22 | 8 | **1.6e-2** | 0.73 | 0.54 | 0.88 |
|  | Juan | 19 | 11 | 0.20 | 0.63 | 0.44 | 0.80 |
|  | Monroe | 28 | 12 | **1.6e-2** | 0.70 | 0.54 | 0.83 |
|  | Vulcain | 24 | 6 | **1.4e-3** | 0.80 | 0.61 | 0.92 |

**Supplementary table 1.** Interspecific differences in the preference task in **B.** the distribution of preferences of which object to removes first **C.** the decision time to remove the first object.

| **Table 1B** glmmTMB(Count of first removals ~ Species + (1\|Subject), Poisson family) | | | | |
| --- | --- | --- | --- | --- |
|  |  | Estimate ± SE | z-value | p-value |
| **Species** | Intercept | -1.73 ± 0.21 | -8.31 | **<0.001 ***** |
|  | Species: Furcifer | 0.56 ± 0.27 | 2.09 | **<0.05 *** |
| **Random effect** | | | | |
|  | Variance | Sd |  | |
| **Subject** | 6.23e-17 | 7.90e-9 |  |  |

| **Table 1C** coxme(Decision time ~ Object removed * Species + (1\|Subject)) | | | | | | | |
| --- | --- | --- | --- | --- | --- | --- | --- |
|  |  | | Coef ± SE | | z-value | | p-value |
| **Object removed** | Object: Stone | | 0.21 ± 0.22 | | 0.98 | | 0.33 |
| **Species** | Species: Furcifer | | 0.87 ± 0.28 | | 3.06 | | **<0.01 **** |
| **Object*Species** | Stone, Furcifer | | -0.43 ± 0.28 | | -1.52 | | 0.13 |
| **Random effect** | | | | | | | |
|  | Variance | | Sd | |  | | |
| **Subject** | 0.19 | | 0.43 | |  |  |  |
| **Type II ANOVA** | | | | | | | |
|  | | Df | | Chi-square | | Pr(>Chisq) | |
| Object removed | | **1** | | 0.10 | | 0.75 | |
| Species | | **1** | | 7.95 | | **<0.01 **** | |
| Object*Species | | **1** | | 2.31 | | 0.13 | |

**PHASE 2: CHOICE AGAINST PREFERENCE TASK**

**SECTION 2: Methods: Number of trials needed to select the stone – statistics**

Two possible outcomes can occur in our choice against preference task: either the fish just keep on selecting objects with the same initial preference they displayed in the preference task (selecting the shell x% of the times and selecting the stone (1-x)% of the times), or they can behave differently and show a change in this preference.

We investigate the first case, in which the selection of the objects only follows the preferences displayed in the preference task, which is our null hypothesis. Each trial can be considered as a Bernoulli trial where selecting the shell is a failure and selecting the stone is a success. We also hypothesize that each test is independent. We can then consider the variable X: “Rank of the success” in each test (i.e., the number of trials needed to select the stone in each test). This variable X follows a geometric distribution of parameter p = x (x between 0 and 1) (a geometric distribution is the probability distribution of the number X of Bernoulli trials needed to get one success; its parameter p is the probability of getting this success in each trial, here we take x because it is the value of the preference shown in the preference task). In such a distribution, the expected value for the number of independent trials to get the first success is $E\left( X \right)= \frac{1}{p}$. It means that in each test, the fish should on average take $\frac{1}{x}$ trials to remove the stone. The probability for a fish to select the stone on trial *k* is $P\left( X=k \right)=x^{k-1}\left( 1-x \right).$ We calculated the number of trials $k$ so that $P\left( X>k \right)<0.05$. This means that there is less than 5% chance that a fish would take $k$ or more trials to select the stone. Thus, if the fish selected objects only according to their initial preference, there would be less than 5% chance to obtain tests with *k* trials or more (in our case, for all individuals, 6 < *k* < 8).

Note that this statistical test is asymmetric as selecting the stone is the endpoint of the experiment. Thus, only tests above *k* trials can make us reject the null hypothesis and make us conclude that the preference for selecting the shell first has increased. For tests below *k* trials, we cannot reject the null hypothesis that the selection choices can be explained by the initial preferences only. In this case, we cannot firmly conclude whether the preference has remained stable compared to phase 1, or if it has shifted towards an increase of preference for the stone, and have to rely on behavioural data that could indicate such a modification.

**SECTION 3: Choice against preference task: overall performance**

**Supplementary table 2.** Performance in the choice against preference task between species, session epoch (beginning and end sessions) and test epoch (beginning and end tests).

| **Table 2** glmmTMB(Number of trials before selecting the stone ~ (Session epoch + Test epoch) * Species + (1\|Subject), Negative binomial family) | | | | | |
| --- | --- | --- | --- | --- | --- |
|  |  | Estimate ± SE | z-value | | p-value |
|  | Intercept | 1.63 ± 0.19 | 8.38 | | **<0.001 ***** |
| **Session epoch** | Epoch: Session 2 | -0.12 ± 0.19 | -0.66 | | 0.51 |
| **Test epoch** | Epoch: End tests | 0.02 ± 0.19 | 0.11 | | 0.91 |
| **Species** | Species: Furcifer | 0.45 ± 0.27 | 1.67 | | 0.09 . |
| **Session*Species** | Session 2*Furcifer | 0.11 ± 0.26 | 0.43 | | 0.67 |
| **Test*Species** | End tests*Furcifer | 0.16 ± 0.26 | 0.61 | | 0.54 |
| **Random effect** | | | | | |
|  | Variance | Sd |  | | |
| **Subject** | 0.06 | 0.25 |  |  |  |
| **Type II ANOVA** | | | | | |
|  |  | Df | Chi square | Pr(>Chisq) | |
| Session epoch | | 1 | 0.25 | 0.61 | |
| Test epoch | | 1 | 0.70 | 0.40 | |
| Species | | 1 | 9.25 | **< 0.01 **** | |
| Session epoch * Species | | 1 | 0.19 | 0.67 | |
| Test epoch * Species | | 1 | 0.38 | 0.54 | |

**SECTION 4: Choice against preference task: behavioural differences**

**Supplementary table 3.** Differences in behaviours during the task depending on the species and/or the object selected **A.** in decision time, **B.** **C. D.** in the number of manipulations of the objects before selecting one (**B.** counts of manipulations before selecting an object, **C.** results of the generalised linear mixed effect model, **D.** post-hoc analyses of the model using the least-square means method).

| **Table 3A** coxme(Decision time ~ Object selected * Species + (1\|Subject)) | | | | |
| --- | --- | --- | --- | --- |
|  |  | Coef ± SE | z-value | p-value |
| **Object selected** | Object: Stone | -0.21 ± 0.14 | -1.56 | 0.12 |
| **Species** | Species: Furcifer | 0.08 ± 0.26 | 0.31 | 0.75 |
| **Object * Species** | Stone*Furcifer | -0.16 ± 0.19 | -0.86 | 0.39 |
| **Random effect** | | | | |
|  | Variance | Sd |  |  |
| **Subject** | 0.18 | 0.43 |  |  |
| **Type II ANOVA** | | | | |
|  | | Df | Chi square | Pr(<Chisq) |
| Object selected | | 1 | 9.44 | **< 0.01 **** |
| Species | | 1 | 0.05 | 0.83 |
| Object selected * Species | | 1 | 0.73 | 0.39 |

| **Table 3B** | | **Object selected** | | TOTAL |
| --- | --- | --- | --- | --- |
|  |  | Shell | Stone |  |
| **Species** | *A. dewindti* | None: 237  Once: 36  Twice: 9  Thrice: 1 | None: 53  Once: 9  Twice: 6  Thrice: 1 | None: 290  Once: 45  Twice: 15  Thrice: 2 |
|  | *C. furcifer* | None: 539  Once: 43  Twice: 6  Thrice: 0 | None: 41  Once: 17  Twice: 4  Thrice: 0 | None: 580  Once: 60  Twice: 10  Thrice: 0 |
| TOTAL | | None: 776  Once: 79  Twice: 15  Thrice: 1 | None: 94  Once: 26  Twice: 10  Thrice: 1 | None: 870  Once: 105  Twice: 25  Thrice: 2 |

| **Table 3C** glmmTMB(Number of manipulations ~ Object selected * Species + (1\|Subject), Negative binomial family) | | | | |
| --- | --- | --- | --- | --- |
|  |  | Estimate ± SE | z-value | p-value |
|  | Intercept | -1.88 ± 0.35 | -5.43 | **<0.001 ***** |
| **Object selected** | Object: Stone | 0.57 ± 0.25 | 2.29 | **<0.05 *** |
| **Species** | Species: Furcifer | -0.80 ± 0.49 | -1.64 | 0.10 |
| **Species*Object** | Furcifer*Stone | 1.02 ± 0.35 | 2.93 | **<0.01 **** |
| **Random effect** | | | | |
|  | Variance | Sd |  |  |
| **Subject** | 0.56 | 0.75 |  |  |
| **Type II ANOVA** | | | | |
|  |  | Df | Chi square | **Pr(<Chisq)** |
| Object selected | | 1 | 38.33 | **< 0.001 ***** |
| Species | | 1 | 0.95 | 0.33 |
| Object selected * Species | | 1 | 8.56 | **< 0.01 **** |

| **Table 3D** | | | **Object selected** | | |
| --- | --- | --- | --- | --- | --- |
|  |  |  | Shell | Stone | |
|  |  |  | *C. furcifer* | *A. dewindti* | *C. furcifer* |
| **Species** | *A. dewindti* | Shell | Estimate = 0.81 ± 0.49  p-value = 0.35 | Estimate = -0.57 ± 0.25  p-value = 0.10 | Estimate = -0.78 ± 0.51  p-value = 0.41 |
|  |  | Stone | Estimate = -1.37 ± 0.51  p-value = **0.04 *** |  | Estimate = -0.22 ± 0.53  p-value = 0.98 |
|  | *C. furcifer* | Shell |  |  | Estimate = -1.59 ± 0.25  p-value = **<0.001***** |
